# Avoidable Deaths--Nursing Practice Failures to Honor Advanced Directives in SNFs--A National Sample

**DOI:** 10.1093/geroni/igaa057.289

**Published:** 2020-12-16

**Authors:** Mary Dellefield, Byron Batz, Catherine Verkaaik, Diane Carter, Caroline Madrigal

**Affiliations:** 1 VA San Diego Healthcare System, San Diego, California, United States; 2 Hahn School of Nursing, Temecula, California, United States; 4 self-employed, Denver, Colorado, United States; 5 Providence VA Medical Center, Providence, Rhode Island, United States

## Abstract

Ongoing evidence of failures to provide cardiopulmonary resuscitation in skilled nursing facilities (SNFs) has resulted in the federal regulation - F678 Cardio-Pulmonary Resuscitation (CPR). Descriptions of CPR-related non-compliance and nursing practice failures are contained in Statements of Deficiencies (CMS-2567). These data provide a unique opportunity to describe practice failures at the point-of-care. A mixed methods case study using content analysis and descriptive statistics was used for a purposeful sample of SODs from 11 SNFs in six states derived from a 2012 first quarter national CMS report of 42 avoidable deaths associated with immediate jeopardy citations. A codebook was developed and tested, based on empirical evidence reported in Office of Inspector General (OIG) 2014 Adverse Events and the Institute of Medicine 2004 nursing surveillance framework. Two trained and independent coders analyzed data. Analysis of SOD quality was conducted. Patterns of practice failures were identified. Ownership included 3 not-for-profit; 1 governmental; and 7 for-profit facilities. The 2012-star ratings ranged from 1.0 – 2.8. The practices of 5 RNs and 5 DONs were described. OIG categories included abuse and neglect, care transitions, and medications. Practice failures were associated with inadequate initiation of CPR resulting from improper processing of orders, poor identification of resident status, poor RN and DON surveillance, absence of CPR certified staff, and a lack of urgency in nursing’s response. The quality of SODs, based on 5 parameters, ranged from 18% - 100%. SODs are useful as data sources. Identified practice failures are useful in developing best practice protocols.

